# Drastic mycorrhizal community shifts in *Sceptridium* ferns during the generation transition from fully mycoheterotrophic gametophytes to photosynthetic sporophytes

**DOI:** 10.1111/nph.20330

**Published:** 2024-12-07

**Authors:** Kenji Suetsugu, Hidehito Okada, Shun K. Hirota, Michimasa Yamasaki, Ryoko Imaichi, Atsushi Ebihara

**Affiliations:** ^1^ Department of Biology, Graduate School of Science Kobe University 1‐1 Rokkodai, Nada‐ku Kobe Hyogo 657‐8501 Japan; ^2^ Institute for Advanced Research Kobe University 1‐1 Rokkodai, Nada‐ku Kobe Hyogo 657‐8501 Japan; ^3^ Botanical Gardens Osaka Metropolitan University 2000 Kisaichi Katano Osaka 576‐0004 Japan; ^4^ Division of Forest and Biomaterials Science, Graduate School of Agriculture Kyoto University Kitashirakawa Oiwake‐cho, Saky Kyoto 606‐8502 Japan; ^5^ Department of Chemical and Biological Sciences, Faculty of Science Japan Women's University Mejirodai Tokyo 112‐8681 Japan; ^6^ Department of Botany National Museum of Nature and Science 4‐1‐1 Amakubo Tsukuba Ibaraki 305‐0005 Japan

**Keywords:** arbuscular mycorrhizal symbiosis, *Entrophospora*, gametophyte, mycoheterotrophy, ontogenetic shift, Ophioglossaceae, sporophyte

## Abstract

- Many plant species experience a prolonged subterranean phase during which they rely entirely on mycorrhizal fungi for carbon. While this mycoheterotrophic strategy spans liverworts, lycophytes, and ferns, most empirical research has centered on angiosperms.
- This study explores the fungal associations of *Sceptridium* (Ophioglossaceae), an early‐diverging fern with mycoheterotrophic gametophytes. We analyzed germination patterns and fungal associations in *Sceptridium* gametophytes, comparing them to the distribution and mycorrhizal partners of photosynthetic sporophytes.
- High‐throughput sequencing data reveal that mycoheterotrophic gametophytes consistently associate with a single *Entrophospora* fungus in the order Entrophosporales (Glomeromycotina), while photosynthetic sporophytes primarily partner with fungi from Glomeraceae (Glomerales, Glomeromycotina). Consequently, gametophytes exhibit spatial clustering without association with adult plants. This is the first documentation of an association between Entrophosporaceae (and the order Entrophosporales) and mycoheterotrophic plants.
- The drastic shifts in *Sceptridium* mycorrhizal communities across life stages likely reflect changing physiological needs during development. Further research is essential to determine whether the association with Entrophosporaceae is widespread among mycoheterotrophic species and to elucidate the functional and physiological mechanisms underlying these mycorrhizal shifts.

## Introduction

Most land plants, from liverworts to angiosperms, engage in mutualistic arbuscular mycorrhizal (AM) symbioses with fungi belonging to Glomeromycotina and, to a lesser extent, Mucoromycotina (Bidartondo *et al*., 2011; van der Heijden *et al*., 2015; Hoysted *et al*., 2018). In these associations, plants acquire essential mineral nutrients from their mycorrhizal partners in exchange for carbon produced through photosynthesis. These AM symbioses are considered pivotal for the colonization and diversification of land plants (Remy *et al*., 1994; Selosse & Le Tacon, 1998; Redecker *et al*., 2000; Wang *et al*., 2010; Hoysted *et al*., 2018). AM associations are the most widespread mycorrhizal interaction, found not only in vascular plants but also in liverworts and hornworts (Wang & Qiu, 2006; Merckx, 2013; Rimington *et al*., 2020), while shifts toward novel mycorrhizal associations with basidiomycete or ascomycete fungi are key innovations that have driven the evolution of specialized life histories and nutritional modes in land plants (Brundrett, 2002; Wang *et al*., 2021).

Many early‐diverging vascular plants, such as lycophytes (the outgroup to all other living vascular plant lineages), exhibit a life cycle characterized by a subterranean mycoheterotrophic gametophyte generation (Winther & Friedman, 2007, 2008, 2009) that produces gametes and, after mating, supports the growth of a diploid, photosynthetic sporophyte. Gametophytes typically rely on Glomeromycotina and Mucoromycotina fungi to meet their carbon requirements (Winther & Friedman, 2007, 2008, 2009; Horn *et al*., 2013; Hoysted *et al*., 2021). This strategy, known as mycoheterotrophy, exploits fungal partners by reversing the usual carbon flow from plant to fungi (Merckx, 2013). This life‐history trait is present in *ca*. one thousand species of ferns and lycophytes across various biomes, from tropical to subalpine ecosystems (Merckx, 2013). Despite its prevalence, information on the associations between mycoheterotrophic gametophytes and AM fungi is relatively scarce, due to the hidden nature of these life stages. Further research is needed to investigate the dynamics of plant–fungal associations throughout the entire life cycle of such plants.

Available evidence indicates that both fully mycoheterotrophic gametophytes and photosynthetic sporophytes of lycophytes (*Lycopodium* and *Huperzia*) and ophioglossoid ferns (*Botrychium* and *Psilotum nudum*) typically exhibit high‐intergenerational fidelity toward a specific clade of Glomeraceae or Mucoromycotina fungi (Winther & Friedman, 2007, 2008, 2009; Perez‐Lamarque *et al*., 2023). This fidelity suggests a ‘take now, pay later’ mechanism in their mycorrhizal relationships, where carbon invested by AM fungal partners to support mycoheterotrophic stages is reciprocated later by green sporophytes (Leake *et al*., 2008; Field *et al*., 2015). This mechanism has also been proposed as the principle underlying fungal specificity and mutualism throughout the mycoheterotrophic‐to‐autotrophic life stages in the development of most green‐leaved orchids (Cameron *et al*., 2008; Read *et al*., 2024). Furthermore, intergenerational fungal specificity may indicate a carbon subsidy from green‐leaved sporophytes to achlorophyllous gametophytes via a shared fungal partner, a form of parental nurture (Leake *et al*., 2008). Indeed, although experimental evidence of this process is still lacking in lycophytes and ferns, recent research shows that the fungal partners of the orchid *Dactylorhiza fuchsii* transfer photosynthates from autotrophic adult plants to mycoheterotrophic protocorms via a common mycorrhizal network, further supporting the parental nurture hypothesis (Read *et al*., 2024).

Ophioglossaceae, along with Psilotaceae, are unique among eusporangiate fern lineages for their fully mycoheterotrophic gametophytes (Zhang *et al*., 2020; Zhang & Zhang, 2022). While the earliest fossils of Ophioglossaceae can only be traced back to the Paleocene (Rothwell & Stockey, 1989), they represent one of the earliest diverging fern clades, with estimated stem ages between 250 and 370 million years (Pryer *et al*., 2004; Rothfels *et al*., 2015; Testo & Sundue, 2016). Ophioglossaceae are distinguishable from other ferns by their bifurcated fronds, which consist of one vegetative leaf and another reproductive, spore‐bearing leaf (Hauk *et al*., 2003). Molecular studies have confirmed the monophyly of the family and identified two species‐rich subfamilies, Botrychioideae and Ophioglossoideae, each containing *ca*. 100 species (Hauk *et al*., 2003; Zhang & Zhang, 2022). Within Botrychioideae, the genera *Botrychium* and *Sceptridium* are species‐rich and nearly cosmopolitan (Zhang & Zhang, 2022).

This study investigates the largely unexplored fungal associates of *Sceptridium*. The taxa studied exhibit typical Ophioglossaceae characteristics: the inconspicuous, subterranean, and Chl‐free gametophyte generation, persisting for several years, is eventually replaced by an erect, green, autotrophic sporophyte (Campbell, 1921; Nozu, 1954; Takahashi & Imaichi, 2007). Although AM associations in these plants have been microscopically observed during their subterranean stages (Daigobo, 1979, 1983), the molecular and phylogenetic identities of the fungal partners throughout the plant life cycle remain unknown, highlighting the need for molecular studies to unravel the fungal partners involved.

In line with previous studies on mycoheterotrophic gametophytes of *Lycopodium*, *Huperzia*, *Botrychium*, and *Psilotum* species (Winther & Friedman, 2007, 2008, 2009), we primarily expect Glomeraceae fungi to be the fungal associates in both gametophyte and sporophyte generations. However, considering dynamic shifts in mycorrhizal partnerships during plant development in certain angiosperms (Jacquemyn & Merckx, 2019; Ventre Lespiaucq *et al*., 2021), we cannot exclude similar mycorrhizal shifts in response to the nutritional or ecological needs of each life stage.

Our primary objectives are to address key questions about mycorrhizal symbiosis in nonseed plants with subterranean mycoheterotrophic stages, specifically focusing on *Sceptridium*: (1) What is the molecular identity of the AM fungi associated with *Sceptridium* species? (2) Do the AM fungal symbionts remain consistent throughout both autotrophic and mycoheterotrophic stages? (3) Is there a spatial association between sporophytes and gametophytes, as predicted by the hypothesis that parental plants act as carbon donors?

## Materials and Methods

### Study species and sites

In *Sceptridium* species, both photosynthetic sporophytes and mycoheterotrophic gametophytes exhibit AM associations, with the dependency on AM fungi being particularly pronounced in the subterranean gametophytes, which rely on these symbionts for growth and gamete production (Campbell, 1921; Nozu, 1954). *Sceptridium* sporophytes generally prefer low‐light environments, with most species inhabiting forest floors (Wagner, 1990; Ebihara, 2016). Many *Sceptridium* species exhibit a winter‐green habit, emerging in fall and persisting into spring, when the tree canopy closes (Cao & Hauk, 2022). This uncommon trait makes *Sceptridium* particularly well‐suited to temperate deciduous forest floors. Although the exact lifespan of *Sceptridium* sporophytes remains undetermined, transplanted individuals have been observed to survive for over 40 yr, indicating notable longevity (A. Ebihara, personal observation).

Field studies were conducted in Tsukuba City, Ibaraki Prefecture (N36.10, E140.11), a region with a warm temperate climate in central Japan. The study site is a plantation dominated by *Cercidiphyllum japonicum*, *Magnolia obovata*, and *Quercus mongolica* var. *crispula*. Several hundred sporophytes of both *Sceptridium japonicum* (Prantl) Lyon and *S. nipponicum* (Makino) Holub naturally grew at the study site during the study years (2009 and 2010). Tsukuba has a mean annual temperature of 14.0°C, with highs averaging 19.2°C, lows of 9.4°C, and annual precipitation of 1242.1 mm (Ministry of Land, Infrastructure, Transport and Tourism, 2024).

To assess the consistency of mycorrhizal associations, we also examined mycorrhizal communities in *S. japonicum* sporophytes (*n* = 9) collected from Kobe City, Hyogo Prefecture (N34.75, E135.12), as well as in both gametophytes and sporophytes of *S. atrovirens* Sahashi (*n* = 4 each) from Ainan Town, Ehime Prefecture (N32.96, E132.58). Kobe has a mean annual temperature of 14.1°C, with an average high of 18.6°C, a low of 10.2°C, and precipitation of 1484.2 mm. Ainan, by contrast, has a mean annual temperature of 16.7°C, with annual highs of 21.4°C, lows of 12.3°C, and annual precipitation of 1936.6 mm (Ministry of Land, Infrastructure, Transport and Tourism, 2024).

### Sporophyte and gametophyte distribution

The survey of sporophyte distribution took place on November 30, December 6, 7, 9, and 10, 2009, in a deciduous broadleaf forest in Tsukuba City, Ibaraki Prefecture. Within this area, *S. japonicum* and *S. nipponicum* were abundantly distributed. A grid consisting of 1 m × 1 m frames within a 17 m by 22 m plot was established to examine sporophyte distribution. Only individuals with vegetative leaves exceeding 3 cm or those bearing reproductive, spore‐bearing leaves were included, and species identification was based on morphology.

Although searching for underground *Sceptridium* gametophytes is often guided by young, emergent sporophytes attached to them, this method overlooks gametophytes that have not yet developed sporophytes (Mason & Farrar, 1989; Johnson‐Groh *et al*., 2002). Therefore, we employed a centrifugation technique following Mason & Farrar (1989) and Johnson‐Groh *et al*. (2002) with slight modifications. In brief, soil samples were collected from 35 sites during 2009 and 2010 using a 5 cm square stainless‐steel corer, providing a uniform volume of *ca*. 100 cm^3^. Each soil sample was disassembled and passed through sieves to remove larger roots and debris. The sediment was mixed with *Sceptridium* root segments stained with neutral red, as a positive control to confirm efficient recovery of the underground parts of *Sceptridium* using the method described below (Mason & Farrar, 1989).

We transferred the sediment into 50 ml centrifuge tubes containing *ca*. 40 ml of water. After stirring, the samples were centrifuged at 2000 rpm (277.51 ***g***) for 2 min, a process repeated twice to isolate live organic matter and stained roots as a pellet at the bottom. This pellet was then suspended in a 50% sucrose solution and centrifuged again at 200 rpm (2.78 ***g***) for 1 min. The resulting supernatant was collected and centrifuged once more at 200 rpm (2.78 ***g***) for an additional minute before filtering through filter paper. The filter paper was placed in a Petri dish, slightly moistened with water, and examined under a Leica M420 stereomicroscope (Leica Microsystems, Wetzlar, Germany) to detect the presence of *Sceptridium* gametophytes. We measured the lengths and widths of each gametophyte using the stereomicroscope to quantify morphological variations. Representative gametophyte samples (*n* > 3 for each species) were hand‐sectioned, stained with 0.05% Trypan Blue, and examined under an Olympus BX‐51 microscope (Olympus, Tokyo, Japan) for morphological analysis of mycorrhizal fungi. Additionally, given the sympatric presence of *S. japonicum* and *S. nipponicum*, and the difficulty in distinguishing the two species based on gametophyte morphology, we identified *Sceptridium* gametophytes collected from the Tsukuba population using MIG‐seq analysis, a type of reduced representation sequencing technique (Supporting Information Methods S1).

To investigate the spatial relationship between sporophytes and gametophytes in *Sceptridium* species, we employed the bivariate O‐ring statistic (Wiegand & Moloney, 2004). This method assessed the expected proximity of sporophyte points to an arbitrary gametophyte point across varying distances (*r*). The null hypothesis was that the spatial distribution of sporophytes was independent of gametophyte locations. To test this hypothesis, simulations were run 99 times by randomly placing the same number of sporophyte points under the assumption of complete spatial randomness (CSR), with 95% confidence intervals estimated. A sporophyte location was considered aggregated around gametophytes if the actual O‐ring statistic at a specific distance was above the upper confidence boundary. A statistic within the confidence envelope indicated a random distribution, while a value below the lower envelope suggested a negative association. These calculations and simulations were performed using the spatstat package (Baddeley *et al*., 2015) in R v.4.2.3 (R Core Team, 2023).

### Molecular analysis of mycorrhizal fungi

The 30 gametophyte samples (26 collected from the Tsukuba population and 4 *S. atrovirens* gametophytes from the Ainan population) and 41 sporophyte samples (15 and 13 *S. japonicum* and *S. nipponicum* sporophytes from the Tsukuba population, 9 *S. japonicum* sporophytes from Kobe, and 4 *S. atrovirens* sporophytes from Ainan) were used for molecular identification of mycorrhizal fungi. After removing potential contaminants, total DNA was extracted using the Qiagen DNeasy® Plant Mini Kit (Qiagen, Valencia, CA, USA).

Given that our morphological investigation indicated AM associations in both gametophytes and sporophytes (see Results), PCR amplification was conducted using the primer set AMV4.5NF/AMDGR, designed primarily to amplify Glomeromycotina fungi (Sato *et al*., 2005), fused with 3–6‐mer Ns. Additional PCR analyses were performed using the FRE‐F/FRE‐R primer set to detect potentially co‐occurring Mucoromycotina fungi (Seeliger *et al*., 2024), but post‐PCR electrophoresis confirmed no amplification with these primers. PCR was performed with the Q5 High‐Fidelity DNA Polymerase kit (New England Biolabs, Ipswich, MA, USA) under the following conditions: initial denaturation at 98°C for 3 min, 35 cycles at 98°C for 10 s, 58°C for 20 s, 72°C for 20 s, and a final extension at 72°C for 10 min. A supplemental PCR was performed to add Illumina P5/P7 adaptor sequences and sample‐specific indices (Syed *et al*., 2009) under these conditions: 98°C for 3 min, followed by 12 cycles at 98°C for 10 s, 65°C for 20 s, and 72°C for 20 s, with a final extension at 72°C for 10 min. Each PCR amplicon was pooled and purified using the AMPure XP Kit (Beckman Coulter, CA, USA). Target‐sized DNA (*c*. 500 bp) was excised using E‐Gel SizeSelect (Thermo Fisher Scientific, Waltham, MA, USA). The pooled library was sequenced on an Illumina MiSeq platform with a MiSeq Reagent Micro Kit v2 (300 cycles, Illumina, USA) at an 8 pM loading concentration, with a 10% PhiX spike‐in. The raw sequencing data have been deposited in the NCBI Sequence Read Archive (SRA accession no. PRJNA1175855).

The data were analyzed using Claident v.0.9.2024.06.10 (Tanabe & Toju, 2013), following Suetsugu & Okada (2021). In brief, primer regions were removed, and low‐quality reads were filtered. Erroneous sequences were denoised using Dada2 (Callahan *et al*., 2016). Potential sequences from PCR chimeras and index‐hopping were eliminated using the clremovechimev and clremovecontam commands in Claident (Esling *et al*., 2015; Nilsson *et al*., 2019). The remaining high‐quality reads were clustered into operational taxonomic units (OTUs) at a 97% similarity threshold using Vsearch v.2.8.0 (Rognes *et al*., 2016). To integrate our findings with broader research on fully mycoheterotrophic plants, each OTU was assigned to virtual taxa (VTXs) in the MaarjAM database (Öpik *et al*., 2010), based on the criteria of sequence similarity of ≥ 90%, a BLAST e‐value of ≤ 1e‐50, and query coverage of ≥ 80% (Wall *et al*., 2020). After discarding rare VTXs (those with one read in each PCR amplicon and those with ≤ 50 reads in all data matrices), differences in VTX richness due to sequencing depth among samples were normalized using an abundance‐based rarefaction method, aligning to a minimum read count of 1687 reads per sample.

### Fungal community analyses

We calculated Shannon‐Wiener and Simpson's diversity indices using the vegan package (Oksanen *et al*., 2015) in R v.4.2.3 (R Core Team, 2023) to assess α‐diversity in each group. These metrics account for species abundance and evenness, with higher values indicating greater diversity (Zhao *et al*., 2021). Variations among groups were assessed using ANOVA and *post hoc* Tukey–Kramer tests in the multcomp package (Hothorn *et al*., 2008).

We also examined AM diversity among samples/groups (β‐diversity) using the Bray–Curtis and Jaccard dissimilarity indices in the vegan package (Oksanen *et al*., 2015). Additionally, β‐diversity was assessed using weighted and unweighted UniFrac distance matrices (WUDM and UDM) to account for the phylogenetic relationships of VTXs, applying the GUniFrac package (Lozupone & Knight, 2005; Lozupone *et al*., 2011; Chen & Chen, 2021). Phylogenetic relationships of VTXs were established using type sequences from the MaarjAM database (Öpik *et al*., 2010). Multiple sequence alignment was performed using Mafft v.7.475 (Katoh & Standley, 2013), and a maximum likelihood (ML) tree was generated with Iq‐Tree 1.6.12 (Nguyen *et al*., 2015) using the TIM2 + F + I + R2 model and 1000 ultrafast bootstraps (Hoang *et al*., 2018). Community‐wide dissimilarity was represented by these matrices, where higher values signify greater phylogenetic heterogeneity. Differences were evaluated using ANOVA in the stats package, and multiple comparisons were performed via the Tukey–Kramer test using the multcomp package.

Nonmetric multidimensional scaling (NMDS) was performed using the metaMDS function in the vegan package. We used the PERMANOVA test via the adonis2 function in the vegan package (10 000 permutations) to identify disparities in mycorrhizal communities among plant groups. Pairwise PERMANOVAs were computed using pairwise.adonis with FDR‐adjusted *P*‐values in the pairwiseadonis package (Martinez Arbizu, 2020).

## Results

### Developmental morphologies of gametophyte and sporophyte

**Fig. 1:**
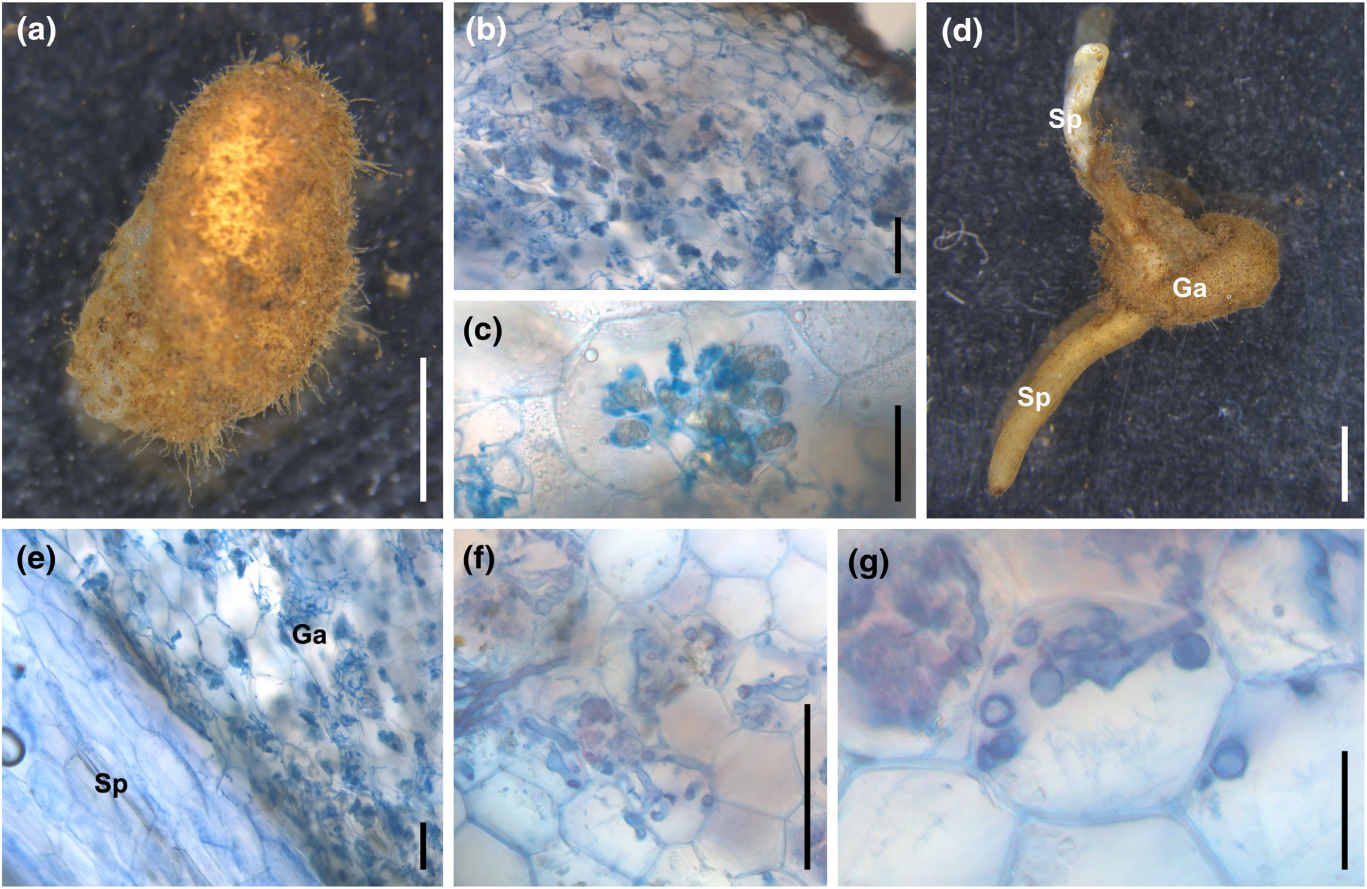
*Sceptridium japonicum* and its mycorrhizal interactions. (a) Gametophyte. (b) Cross‐section of the gametophyte. (c) Close‐up of a fungal coil within the gametophyte. (d) Juvenile sporophyte with the gametophyte. (e) Cross‐section of the gametophyte–sporophyte interface. (f) Cross‐section of a root of a young sporophyte bearing only a vegetative leaf. (g) Close‐up of a fungal coil within the roots of the sporophyte. ‘Ga’ indicates the gametophyte, and ‘Sp’ indicates the sporophyte. The fungal coils were stained with trypan blue. Bars: (a, d) 2 mm; (b, e, f) 200 μm; (c, g) 50 μm.

The gametophyte has a subterranean, tuberous structure that is horizontally flattened (Fig. 1a), with a central dorsal ridge where spermatozoid‐maturing antheridia develop. Juvenile stages, which lack a fully developed ridge, are covered with numerous elongated rhizoids that become less prominent as the gametophyte matures. A longitudinal section of a young gametophyte reveals an apical region containing potential meristematic cells. Fungal partners form *Paris*‐type associations, characterized by intracellular hyphal coils and cell‐to‐cell spread, which are absent in meristematic and reproductive regions. Older hyphal coils often exhibit apex swelling and eventually degenerate into amorphous clumps (Fig. 1b,c). In older gametophytes, the dorsal region lacks fungal symbionts, while the ventral region shows fungal proliferation.

The sporophyte generation of *Sceptridium* is initially parasitic on the gametophyte until its tissues emerge above the substrate and into the light (Fig. 1d). No direct intergenerational transmission of mycorrhizal fungi occurs through the gametophyte–sporophyte interface, as fungi appear excluded from this connection (Fig. 1e). Instead, sporophytes independently establish mycorrhizal associations from their first root. In well‐developed photosynthetic sporophytes, multiple two‐ to four‐cell layers of the root cortex are colonized by *Paris*‐type AM‐forming hyphae (Fig. 1f,g). Degenerated fungal material was observed in some cells. Microscopic analysis revealed coarse intracellular hyphal coils characteristic of Glomeromycotina. However, hyphal coils in *Sceptridium* gametophytes are generally finer than those observed in sporophytes, despite the presence of large vesicles typically associated with Glomeromycotina (Walker *et al*., 2018; Ogura‐Tsujita *et al*., 2019; Prout *et al*., 2024).

### Sporophyte and gametophyte distribution

**Fig. 2:**
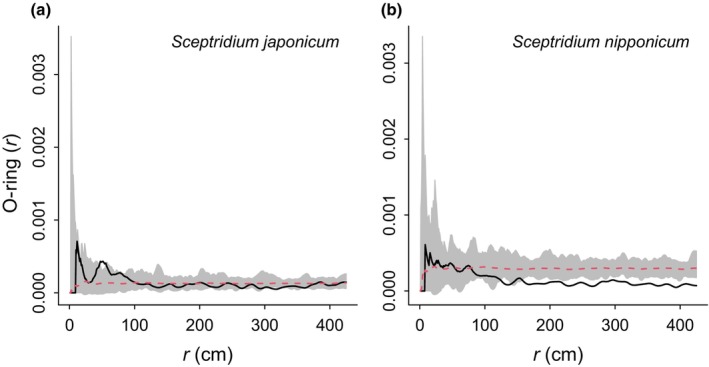
Results of bivariate O‐ring statistics showing the spatial relationships between *Sceptridium japonicum* (a), *Sceptridium nipponicum* (b), and their gametophytes at the Tsukuba population. In the graphical representations, solid lines represent the estimated statistics, interpreted as the density of sporophytes occurring within a ring of radius (*r*). Grey polygons delineate the 95% confidence intervals derived from 99 simulations of points under complete spatial randomness (CSR), with dashed red lines indicating the simulation means.

A total of 1103 *S. nipponicum* sporophytes and 486 *S. japonicum* sporophytes were recorded at the Tsukuba population. Field observations indicated no spatial association between *Sceptridium* sporophytes and gametophytes. Sporophytes were observed throughout the surveyed area, while gametophytes were confined to limited locations, detected in only 7 out of the 35 examined sites. These gametophytes were not necessarily situated in areas with abundant sporophytes (Fig. S1). O‐ring statistic analyses confirmed no significant spatial aggregation between sporophytes and gametophyte locations for *S. japonicum* and *S. nipponicum* (Fig. 2). For *S. japonicum*, calculated statistics generally fell within the confidence interval, indicating a random distribution of sporophytes around gametophytes. Similar results were observed for *S. nipponicum*, with a negative association detected at distances greater than 150 cm from the gametophytes.

Using MIG‐seq data, maximum likelihood phylogenetic trees clearly distinguished the two *Sceptridium* species with 100% bootstrap support (Fig. S2). Neighbor‐Net and ADMIXTURE analyses further corroborated this separation (Figs S3, S4). Molecular data showed most gametophytes belonged to *S. nipponicum* (*n* = 23), while *S. japonicum* represented a smaller fraction (*n* = 3). This composition likely reflects the aboveground sporophyte abundance. Gametophytes from different species coexisted in the same confined space and soil layer, suggesting a lack of spatial segregation. Interestingly, Admixture analysis suggested a potential genetic mixture in one gametophyte (namely, eb81), with a dominant contribution from *S. nipponicum* and a minor contribution from *S. japonicum*. Given that other phylogenetic analyses did not strongly support a hybrid status (e.g. no intermediate positioning in Neighbor‐Net analysis), it was tentatively classified as *S. nipponicum*. However, the lack of spatial and mycorrhizal segregation between these species (see next section) may facilitate interspecific fertilization, and thus, the possibility of a hybrid status for this gametophyte cannot be entirely ruled out.

### Mycorrhizal shifts between gametophytes and sporophytes

**Fig. 3:**
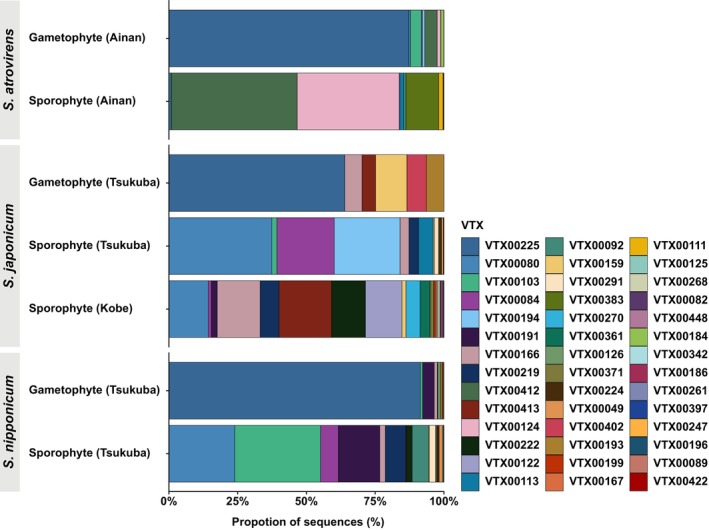
Relative abundance of mycorrhizal communities associated with the *Sceptridium* species investigated.

**Fig. 4:**
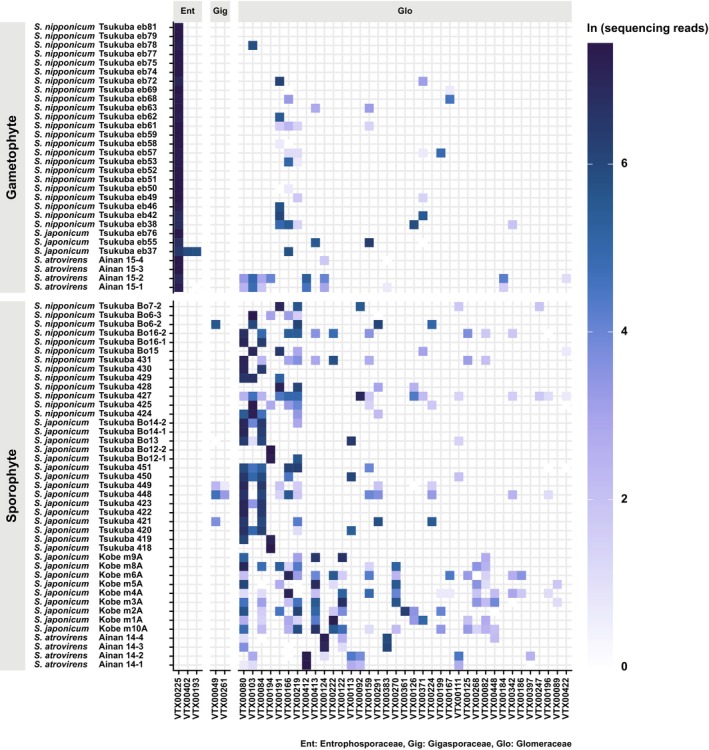
Interaction matrix between the *Sceptridium* species investigated and arbuscular mycorrhizal fungi virtual taxa (VTXs). Color intensity reflects the relative abundance of sequencing reads.

High‐throughput sequencing data revealed that mycoheterotrophic gametophytes predominantly associate with a single *Entrophospora* (formerly *Claroideoglomus*) VTX (VTX00225; 44 583 reads, accounting for 88.09% of all reads in gametophyte samples) within the order Entrophosporales (Glomeromycotina). Entrophosporales, a recently established order, is positioned as a sister group to the combined clade of Glomerales and Diversisporales (Glomeromycotina; Błaszkowski *et al*., 2022). Searches in the MaarjAM database showed that VTX00225 is widespread across diverse anthropogenic and natural ecosystems on multiple continents, primarily in temperate regions (Öpik *et al*., 2010). Notably, this VTX was consistently detected in all gametophyte samples across species and localities (Figs 3, 4, S5). Although (1) gametophytes occasionally harbor additional VTXs and (2) these VTXs often overlap with those found in sporophytes, the lack of a clear pattern between other VTXs and gametophyte size (Fig. S6) suggests that these fungi are likely acquired randomly during the gametophyte generation, rather than exclusively during later stages of gametophyte development.

Photosynthetic sporophytes associate with a diverse range of VTXs, although they show a preference for VTXs from the family Glomeraceae (Glomerales, Glomeromycotina). A total of 38 VTXs (69 167 reads) were identified in their mycorrhizal samples, with 36 VTXs classified as Glomeraceae (68 717 reads, 99.35% of all reads in sporophyte samples) and 2 VTXs classified as Gigasporaceae (450 reads, 0.65%) (Table S1). Notably, VTX00225 was absent from sporophytes across all species (Figs 4, S5). Many VTXs observed in *Sceptridium* sporophytes are also associated with fully mycoheterotrophic species. For example, VTX00080 – a dominant mycorrhizal fungus in *S. nipponicum* and *S. japonicum* sporophytes – is associated with various fully mycoheterotrophic species across Japan, tropical Southeast Asia, South America, and Africa (Suetsugu & Okada, 2021; Suetsugu *et al*., 2022).

**Fig. 5:**
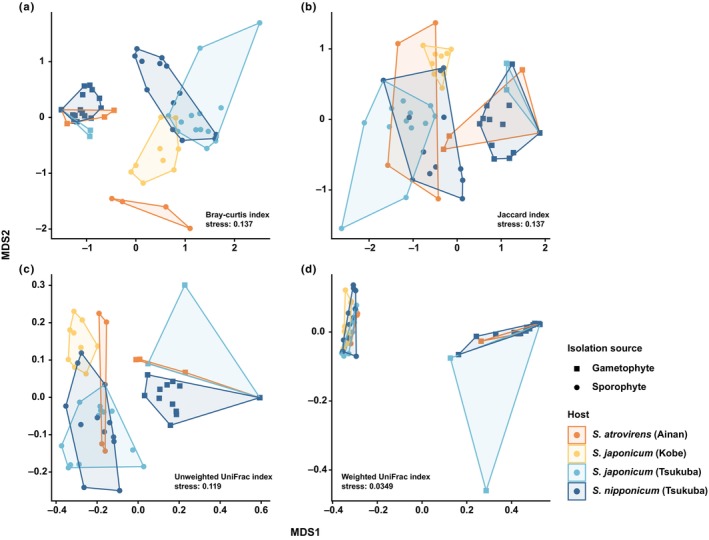
Nonmetric multidimensional scaling (NMDS) plots showing mycorrhizal communities of the *Sceptridium* species investigated, based on (a) Bray–Curtis dissimilarity index, (b) Jaccard dissimilarity index, (c) unweighted UniFrac distance matrix (WUDM), and (d) weighted UniFrac distance matrix (UDM).

The fungal α‐diversity (measured using the Shannon‐Wiener and Simpson's diversity indices) of the AMF communities was not often significantly lower in the gametophyte generation compared to the sporophyte generation. Specifically, significant differences in the Shannon‐Wiener index were observed in only 4 out of 21 comparisons, with diversity consistently higher in the sporophyte generation (Table S2). Similarly, significant differences in Simpson's diversity index were detected in only 3 out of 21 comparisons, again showing higher diversity in the sporophyte generation (Table S3). However, high α‐diversity does not necessarily indicate a lack of specialization, as it can occur when a plant species specializes in key mycorrhizal partners that provide unique resources, alongside additional nonspecific partners offering less critical resources (Shefferson *et al*., 2019). Actually, analyses of fungal β‐diversity, based on Bray–Curtis and Jaccard dissimilarity indices as well as WUDM and UDM, showed that dissimilarity indices between *S. nipponicum* gametophytes were almost always significantly lower than those between *S. nipponicum* sporophytes. Similarly, dissimilarity indices between *S. japonicum* gametophyte samples were almost always significantly lower than those between *S. japonicum* sporophyte samples (*P* < 0.01; Table S4). This indicates that gametophyte fungal associations are more consistent than those of sporophytes. NMDS plot analysis further distinguished AMF communities between the gametophytes and sporophytes, with gametophyte communities clustering closely, even across different species (Fig. 5; Table S5).

## Discussion

### Novel mycoheterotrophic interaction between *Entrophospora* and *Sceptridium*

We identified a consistent mycorrhizal association between fully mycoheterotrophic *Sceptridium* gametophytes and a single *Entrophospora* species (VTX00225). While AM α‐diversity was not significantly lower during the gametophyte generation, specialization can still occur when a plant primarily relies on key mycorrhizal partners, with additional nonspecific fungi providing less critical resources (Shefferson *et al*., 2019; Suetsugu *et al*., 2021). Given the consistently high abundance of VTX00225 across all gametophyte samples, we consider *Sceptridium* gametophytes to be specialized on this fungus. Although the fine hyphae and terminal hyphal swelling observed in *Sceptridium* gametophytes do not entirely exclude the possibility of co‐colonization by Mucoromycotina (e.g. Rimington *et al*., 2015, 2020; Perez‐Lamarque *et al*., 2023), Mucoromycotina fungi are unlikely to be major mycobionts due to the lack of amplification with Mucoromycotina‐specific primers. Therefore, the observed morphological differences in mycorrhizal structures between *Sceptridium* sporophytes and gametophytes likely reflect differences in fungal partners within Glomeromycotina (Glomeraceae vs Entrophosporaceae) and the degree of mycoheterotrophy (at least partial autotrophy vs full mycoheterotrophy).

Our findings generally support the trend that mycoheterotrophic plant species tend to specialize in ‘narrow’ clades of fungi (Bidartondo *et al*., 2002; Yamato *et al*., 2014; Gomes *et al*., 2017). Nonetheless, most mycorrhizal fungi identified in AM‐forming mycoheterotrophic plants belong to the family Glomeraceae, regardless of plant phylogenetic background (Bidartondo *et al*., 2002; Merckx *et al*., 2012; Suetsugu *et al*., 2014; Yamato *et al*., 2014; Gomes *et al*., 2017; Perez‐Lamarque *et al*., 2020; Zhao *et al*., 2021). For example, Yamato *et al*. (2014) found that the mycorrhizal fungi associated with the fully mycoheterotrophic *Petrosavia sakuraii* belonged to a single clade within Glomeraceae, while its autotrophic sister species *Japonolirion osense* associated with a more diverse range of mycorrhizal fungi, including Glomeraceae, Acaulosporaceae, and Diversisporaceae. Similarly, Zhao *et al*. (2021) demonstrated a gradual shift away from non‐Glomeraceae fungi during the mycoheterotrophic evolution of *Burmannia* species with different trophic modes. Notably, VTX00225, which is predominantly associated with *Sceptridium* gametophytes, has not been identified in other mycoheterotrophic lycophytes (*Lycopodium* and *Huperzia*) or ferns (*Botrychium* and *Psilotum nudum*). Furthermore, no previous associations between Entrophosporaceae (or the order Entrophosporales) and mycoheterotrophic plants have been documented (Perez‐Lamarque *et al*., 2020). This newly identified partnership broadens our understanding of the diversity of fungal partners in mycoheterotrophic plants.

Genomic studies have positioned Entrophosporaceae as a sister group to a clade that includes Glomeraceae, Diversisporaceae, and Acaulosporaceae (Perez‐Lamarque *et al*., 2022). Interestingly, these early‐diverging clades, including Entrophosporaceae, are often perceived as less beneficial to plants compared to more rapidly diversifying groups such as Glomeraceae and Diversisporaceae (Säle *et al*., 2021). In a biological marketplace model of mycorrhizal interactions, these early‐diverging fungi might be less effective in maintaining beneficial symbioses with autotrophic plants, potentially making them less preferred partners (Kiers *et al*., 2011). However, their potentially less stringent control over nutritional exchanges might render them ideal for mycoheterotrophic plants, particularly in certain environments. Since these fungi may form parasitic associations with autotrophic AM plants (Säle *et al*., 2021), they could discriminate less in their interactions, making them more vulnerable to exploitation by mycoheterotrophic plants.

Mycoheterotrophic plants tend to favor keystone fungi, which connect them to a wide variety of autotrophic plants, offering a more stable and reliable carbon source (Gomes *et al*., 2022). Notably, the AM taxon utilized by mycoheterotrophic *Sceptridium* gametophytes (VTX00225) is a keystone taxon with high‐network connectivity in certain low‐disturbance forests (Wall *et al*., 2020), where the gametophytes thrive. It also displays resilience in agricultural and urban environments, including lawns and gardens, forming mycorrhizal associations with a broad range of plant taxa, such as species in Poaceae, Rosaceae, and Lamiaceae (Öpik *et al*., 2010). Additionally, *Entrophospora* fungi, including VTX00057 and VTX00193, have been identified in photosynthetic gametophytes of certain ferns, such as *Osmunda banksiifolia* (Osmundaceae) and *Plagiogyria euphlebia* (Plagiogyriaceae) (Ogura‐Tsujita *et al*., 2013). Considering the limited studies on fungal associates in mycoheterotrophic fern gametophytes (Winther & Friedman, 2007, 2009), further research will be essential to evaluate the ecological role of *Entrophospora* fungi in other ferns with mycoheterotrophic gametophytes.

### Intergenerational mycorrhizal community shifts in *Sceptridium*

Our research reveals a notable shift in mycorrhizal associations in *Sceptridium* species during the transition from mycoheterotrophic gametophytes to autotrophic sporophytes. Gametophytes predominantly associate with a single *Entrophospora* VTX (VTX00225), while sporophytes primarily engage with Glomeraceae VTXs. Consequently, *Sceptridium* gametophytes were often clustered but did not spatially align with adult sporophytes.

In many mycoheterotrophic plants, the availability of appropriate mycorrhizal fungi is crucial for seedling recruitment (McCormick & Jacquemyn, 2014). Previous studies often report a strong correlation between adult plants and seed germination, attributed to consistent mycorrhizal partnerships throughout the life cycle (Diez, 2007; Jacquemyn *et al*., 2007, 2012; Waud *et al*., 2016; McCormick *et al*., 2018). Adult autotrophic plants of initially mycoheterotrophic species host dense fungal networks, likely facilitating carbon transfer to nearby seedlings, which may explain why seedling recruitment predominantly occurs near parent plants (Waud *et al*., 2016; McCormick *et al*., 2018; Read *et al*., 2024). However, all currently known examples of such correlations are from orchid mycorrhizas with basidiomycetes. Our study found no significant correlation between the abundance of gametophytes and sporophytes, likely due to the different mycorrhizal preferences between these two life stages. Adult plants primarily associate with Glomeraceae fungi, which are nearly absent in gametophytes. Therefore, proximity to sporophytes does not enhance the availability of necessary mycorrhizal fungi for gametophyte formation.

Previous research indicates a high fidelity of specific arbuscular mycorrhizal fungi with sporophytes and gametophytes in *Botrychium* (Ophioglossaceae), *Huperzia* (Lycopodiaceae), and *Psilotum* (Psilotaceae) (Winther & Friedman, 2007, 2008, 2009). This fidelity is thought to support parental nurture, where organic carbon is transferred across generations (Winther & Friedman, 2007, 2008, 2009; Leake *et al*., 2008; Field *et al*., 2015). Cameron *et al*. (2008) proposed a ‘give now, get more later’ model in green orchids, where fungi invest resources in the early mycoheterotrophic stage, which is later reciprocated when the plant becomes autotrophic. However, given the observed mycorrhizal shift, neither of these concepts applies to *Sceptridium* species and VTX00225. The lack of direct intergenerational transmission of mycorrhizal fungi through the gametophyte–sporophyte interface may favor different mycorrhizal associations. However, this alone does not explain the shift, as independent recruitment of the same partners has been observed in *Botrychium* and *Huperzia*, both of which maintain similar mycorrhizal communities across stages (Leake *et al*., 2008).

An intriguing aspect is the complete absence of *Entrophospora* in mature *Sceptridium* sporophytes. Such drastic transitions are rare among mycoheterotrophic plants, with exceptions including the orchids *Gastrodia elata* (Chen *et al*., 2019), *G. confusoides* (Li *et al*., 2022), and the Ericaceae species *Pyrola asarifolia* (Hashimoto *et al*., 2012). Drastic changes in mycorrhizal communities can create challenges in acquiring new partners and may negatively impact the population dynamics of mycoheterotrophs (Ogura‐Tsujita *et al*., 2021; Ventre Lespiaucq *et al*., 2021). Therefore, partner switching likely occurs only when it confers advantages in resource acquisition. For instance, in the fully mycoheterotrophic orchid *G. elata*, a shift from litter‐decaying *Mycena* fungi to *Armillaria* fungi allows access to a larger carbon pool (Ogura‐Tsujita *et al*., 2021).

The shifts in *Sceptridium* species during the generational transition may reflect changes in resource acquisition strategies similar to mycorrhizal transitions observed in some orchids that become autotrophic upon reaching adulthood (Bayman *et al*., 2016; Zahn *et al*., 2022). If *Sceptridium* sporophytes are fully autotrophic, transitioning to conventional mutualistic mycorrhizal associations, fungi such as *Entrophospora* sp., which are less beneficial for autotrophic plants (Säle *et al*., 2021), would not effectively provide minerals. Consequently, sporophytes may have evolved associations with fungi that better meet their nutrient demands. Alternatively, if *Sceptridium* sporophytes retain some level of mycoheterotrophy, as shown in some Ophioglossaceae species (e.g. Suetsugu *et al*., 2020), their larger biomass might require greater carbon input from fungal partners, despite their partial autotrophy. This hypothesis may be indirectly supported by the fact that most VTXs found in *Sceptridium* sporophytes (e.g. VTX00080 and VTX00084) largely overlap with those in fully mycoheterotrophic species across multiple families, including Burmanniaceae, Gentianaceae, Petrosaviaceae, Polygalaceae, Thismiaceae, and Triuridaceae (Perez‐Lamarque *et al*., 2020; Suetsugu & Okada, 2021; Suetsugu *et al*., 2022). Overall, regardless of the specific nature of these associations, shifts in plant physiological requirements likely drive the observed changes in mycorrhizal partners.

### Conclusion

We revealed a drastic shift in fungal partners between the mycoheterotrophic gametophytes and photosynthetic sporophytes in *Sceptridium* species. The interaction between Entrophosporaceae and mycoheterotrophic plants represents a novel discovery. Whether this is a unique case or a frequent but undocumented phenomenon among other mycoheterotrophs, particularly gametophytes, remains uncertain. Future studies, including ancestral character‐state reconstruction analyses with more extensive mycorrhizal data from other Ophioglossaceae species, will be crucial for understanding the coevolution between ferns and AM fungi.

The dramatic change in mycorrhizal associations in *Sceptridium* species is potentially linked to physiological transitions. Future research should focus on the functional and physiological mechanisms driving this shift. Experimental studies under controlled conditions could provide key insights into the symbiotic interactions and resource translocation dynamics between these partners.

## Competing interests

None declared.

## Author contributions

KS designed the study and wrote the initial draft of the manuscript. AE, RI and KS collected materials. KS, AE and SKH conducted the laboratory experiments. KS, HO, SKH and MY performed the analyses. All authors revised the manuscript and approved the final version.

## Disclaimer

The New Phytologist Foundation remains neutral with regard to jurisdictional claims in maps and in any institutional affiliations.

## Supporting information


**Fig. S1** Spatial distribution of *Sceptridium japonicum* and *S. nipponicum* sporophyte individuals at the Tsukuba population, including information on soil sampling points.
**Fig. S2** Maximum likelihood phylogenetic tree of the *Sceptridium* gametophyte individuals, reconstructed from MIG‐seq data.
**Fig. S3** Neighbor‐Net network of the *Sceptridium* gametophyte individuals, based on uncorrected P distances from MIG‐seq data.
**Fig. S4** Population structure of the *Sceptridium* gametophyte individuals, inferred using ADMIXTURE.
**Fig. S5** Relative abundance of mycorrhizal communities associated with each *Sceptridium* individual.
**Fig. S6** Relative abundance of mycorrhizal communities associated with each *Sceptridium* gametophyte, including information on its length.
**Methods S1** Molecular identification of *Sceptridium* gametophytes.

**Table S1** Sequencing reads of arbuscular mycorrhizal fungi virtual taxa (VTXs) detected in each sample after coverage‐based rarefaction.
**Table S2** Multiple comparisons of fungal α‐diversity based on the Shannon‐Wiener index.
**Table S3** Multiple comparisons of fungal α‐diversity based on the Simpson's diversity index.
**Table S4** Multiple comparisons of fungal β‐diversity based on Bray–Curtis and Jaccard dissimilarity indices, as well as WUDM and UDM.
**Table S5** Results of pairwise PERMANOVA tests for fungal compositional differences based on Bray–Curtis and Jaccard dissimilarity indices, as well as WUDM and UDM.
Please note: Wiley is not responsible for the content or functionality of any Supporting Information supplied by the authors. Any queries (other than missing material) should be directed to the *New Phytologist* Central Office.

## Data Availability

MIG‐seq data and fungal community data have been deposited in the Sequence Read Archive under accession numbers PRJDB19087 and PRJNA1175855, respectively.
