# Supplementary material for: Drastic mycorrhizal community shifts in Sceptridium ferns during the generation transition from fully mycoheterotrophic gametophytes to photosynthetic sporophytes
Source: New Phytol. 2024 Dec 7;245(4):1705–17. doi: 10.1111/nph.20330 (PMC11754932; doi:10.1111/nph.20330)
Supplement: Supplementary file 1 — Fig. S1 Spatial distribution of Sceptridium japonicum and S. nipponicum sporophyte individuals at the Tsukuba population, including information on soil sampling points. Fig. S2 Maximum likelihood phylogenetic tree of the Sceptridium gametophyte individuals, reconstructed from MIG‐seq data. Fig. S3 Neighbor‐Net network of the Sceptridium gametophyte individuals, based on uncorrected P distances from MIG‐seq data. Fig. S4 Population structure of the Sceptridium gametophyte individuals, inferred using ADMIXTURE. Fig. S5 Relative abundance of mycorrhizal communities associated with each Sceptridium individual. Fig. S6 Relative abundance of mycorrhizal communities associated with each Sceptridium gametophyte, including information on its length. Methods S1 Molecular identification of Sceptridium gametophytes. [file NPH-245-1705-s001.pdf]

New Phytologist **Supporting Information**

Article title: **Drastic mycorrhizal community shifts in *Sceptridium* ferns during the generation transition from fully mycoheterotrophic gametophytes to photosynthetic sporophytes**

Authors: Kenji Suetsugu, Hidehito Okada, Shun K. Hirota, Michimasa Yamasaki, Ryoko Imaichi, Atsushi Ebihara

Article acceptance date: 19 November 2024

The following Supporting Information is available for this article:

Fig. S1 Spatial distribution of *Sceptridium japonicum* and *S. nipponicum* sporophyte individuals at the Tsukuba population, including information on soil sampling points.

Fig. S2 Maximum likelihood phylogenetic tree of the *Sceptridium* gametophyte individuals, reconstructed from MIG-seq data.

Fig. S3 Neighbor-Net network of the *Sceptridium* gametophyte individuals, based on uncorrected P distances from MIG-seq data.

Fig. S4 Population structure of the *Sceptridium* gametophyte individuals, inferred using ADMIXTURE.

Fig. S5 Relative abundance of mycorrhizal communities associated with each *Sceptridium* individual.

Fig. S6 Relative abundance of mycorrhizal communities associated with each *Sceptridium* gametophyte, including information on its length.

Table S1 Sequencing reads of arbuscular mycorrhizal fungi virtual taxa (VTXs) detected in each sample after coverage-based rarefaction (separate file).

Table S2 Multiple comparisons of fungal α-diversity based on the Shannon–Wiener index (separate file).

Table S3 Multiple comparisons of fungal α-diversity based on the Simpson's diversity index (separate file).

Table S4 Multiple comparisons of fungal β-diversity based on Bray-Curtis and Jaccard dissimilarity indices, as well as WUDM and UDM (separate file).

Table S5 Results of pairwise PERMANOVA tests for fungal compositional differences based on

Bray-Curtis and Jaccard dissimilarity indices, as well as WUDM and UDM (separate file).

Method S1 Molecular identification of *Sceptridium* gametophytes.

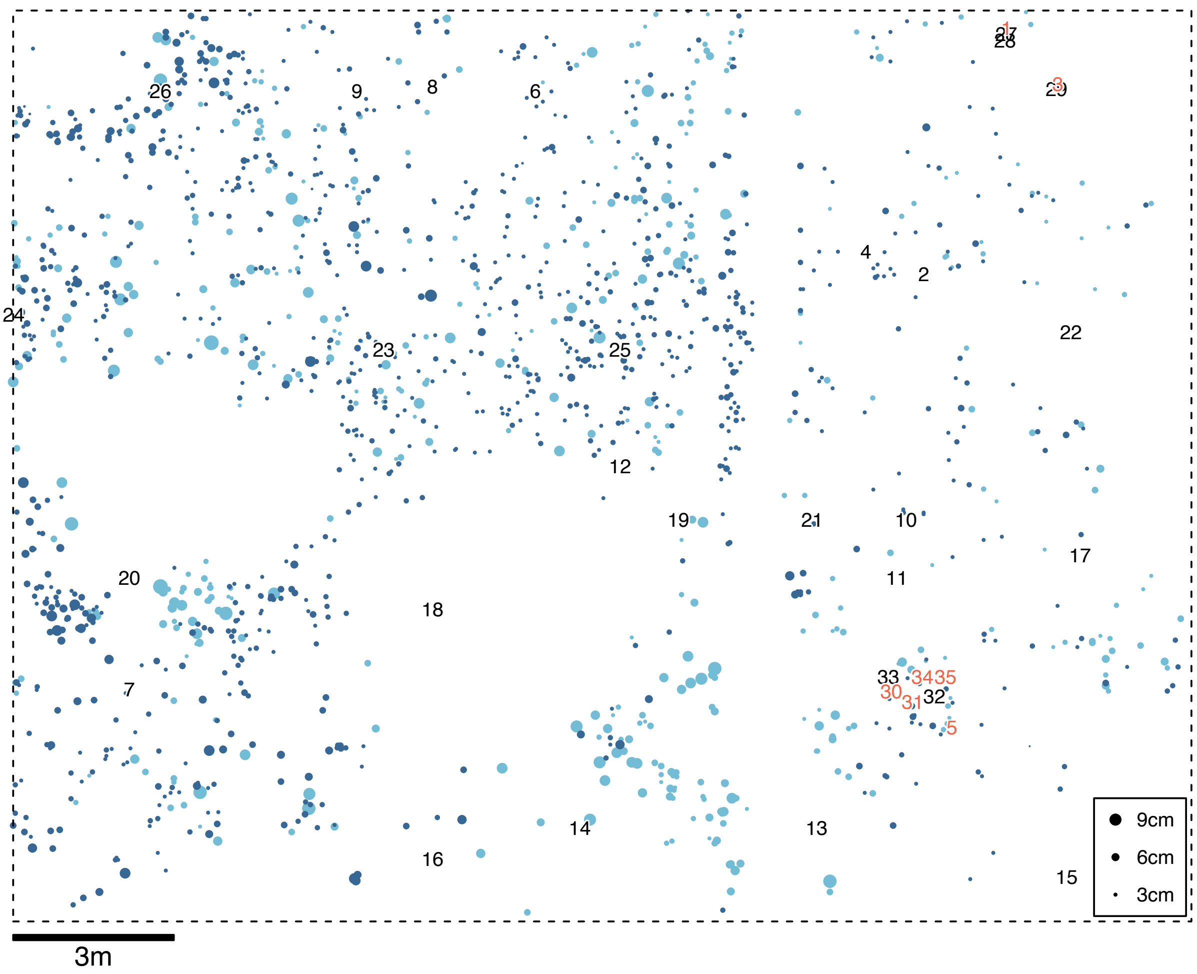

Figure S1. Spatial distribution of *Sceptridium japonicum* (dark blue circles) and *S. nipponicum* (pale blue circles) sporophyte individuals at the Tsukuba population, with point sizes corresponding to trophophore lengths. Soil sampling points are numbered, with red numerals indicating locations where *Sceptridium* gametophytes were discovered.

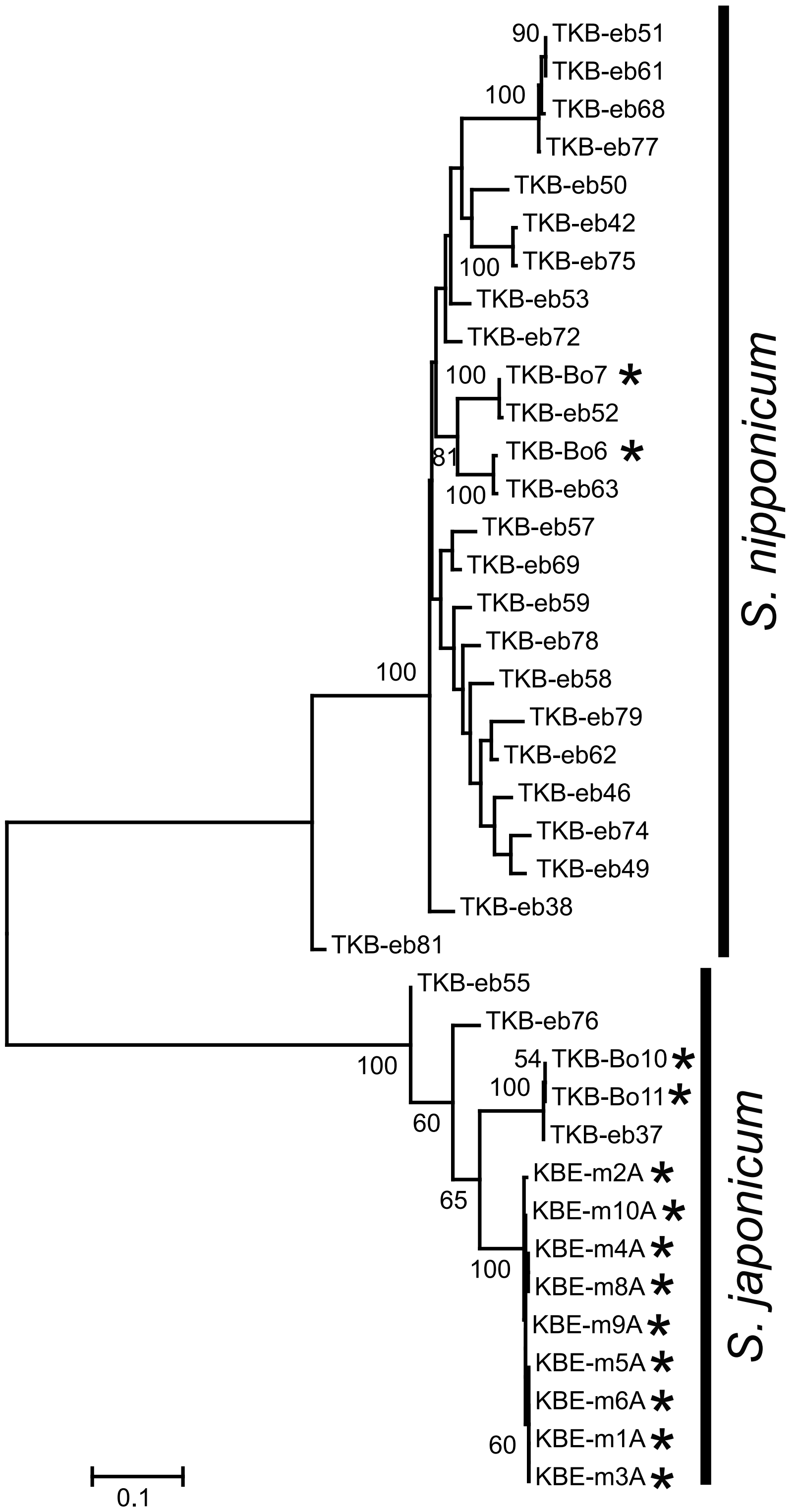

Figure S2. Maximum likelihood phylogenetic tree of the *Sceptridium* gametophyte individuals, reconstructed from MIG-seq data. Nodes supported by bootstrap values <50% are not shown. Branch lengths represent the average number of substitutions per site. Sporophyte samples (controls for gametophyte identification) are marked with an asterisk. Details of the sample IDs are included in Table S1.

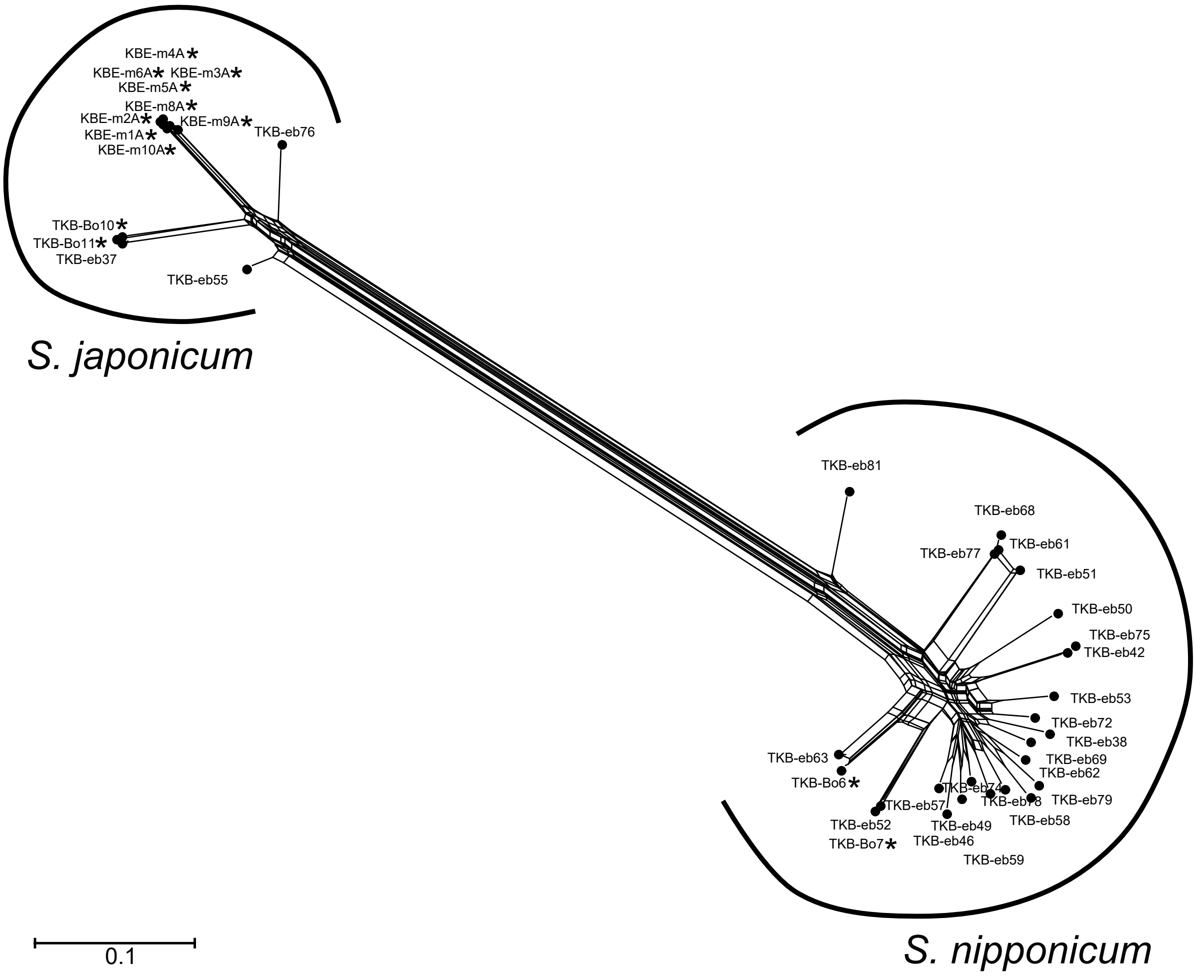

Figure S3. Neighbor-Net network of the *Sceptridium* gametophyte individuals, based on uncorrected *P* distances from MIG-seq data. Sporophyte samples (controls for gametophyte identification) are marked with an asterisk. Details of the sample IDs are included in Table S1.

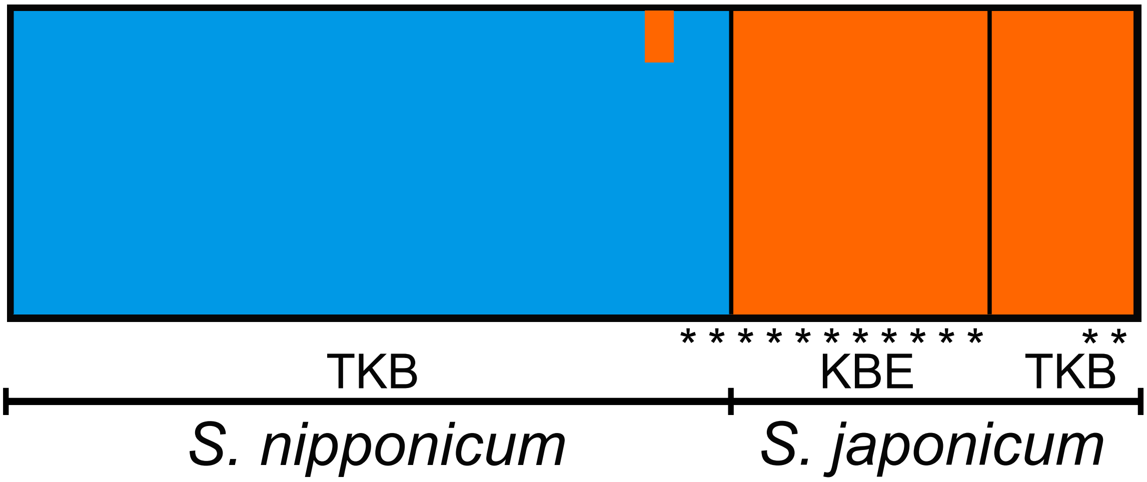

Figure S4. Population structure of the *Sceptridium* gametophyte individuals, inferred using ADMIXTURE. Based on cross-validation error, K = 2 was selected as the optimal model. Populations are separated by vertical black lines. Sporophyte samples (controls for gametophyte identification) are marked with an asterisk.

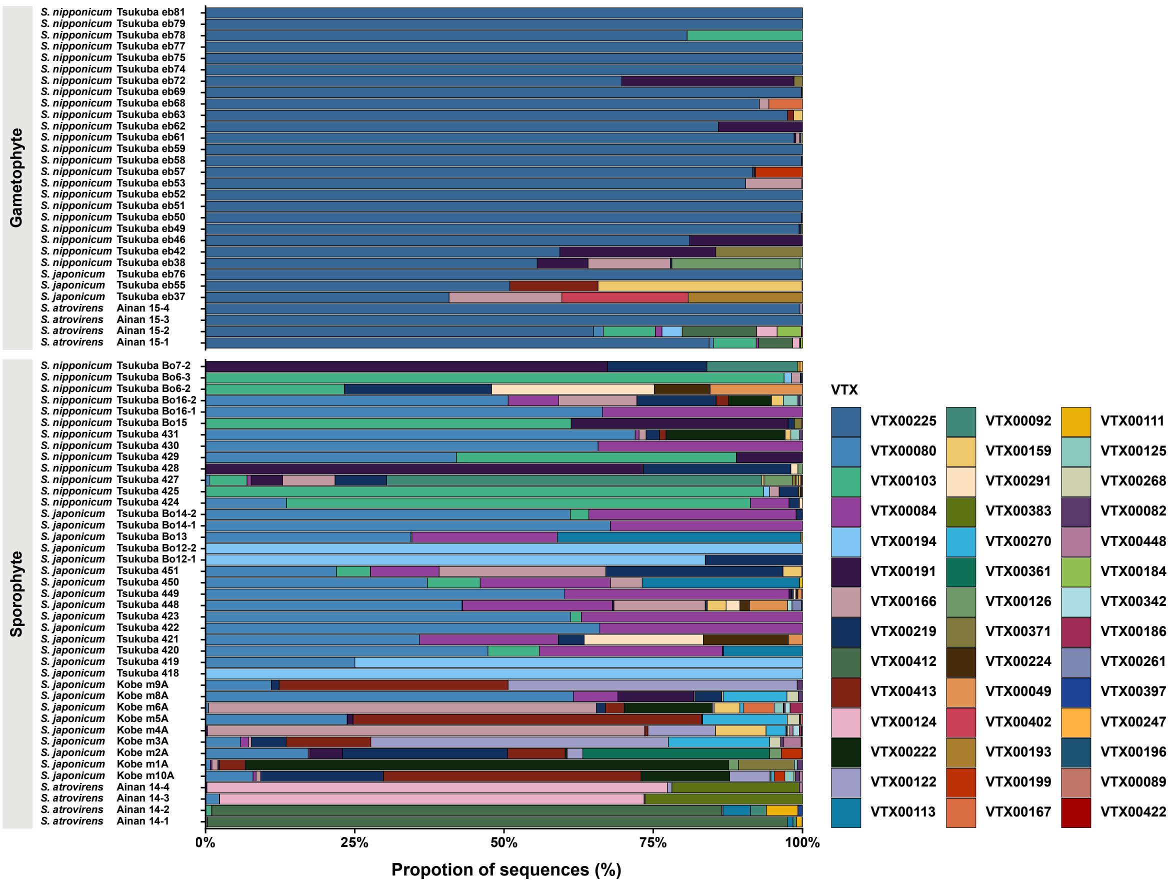

Figure S5. Relative abundance of mycorrhizal communities associated with each *Sceptridium* individual.

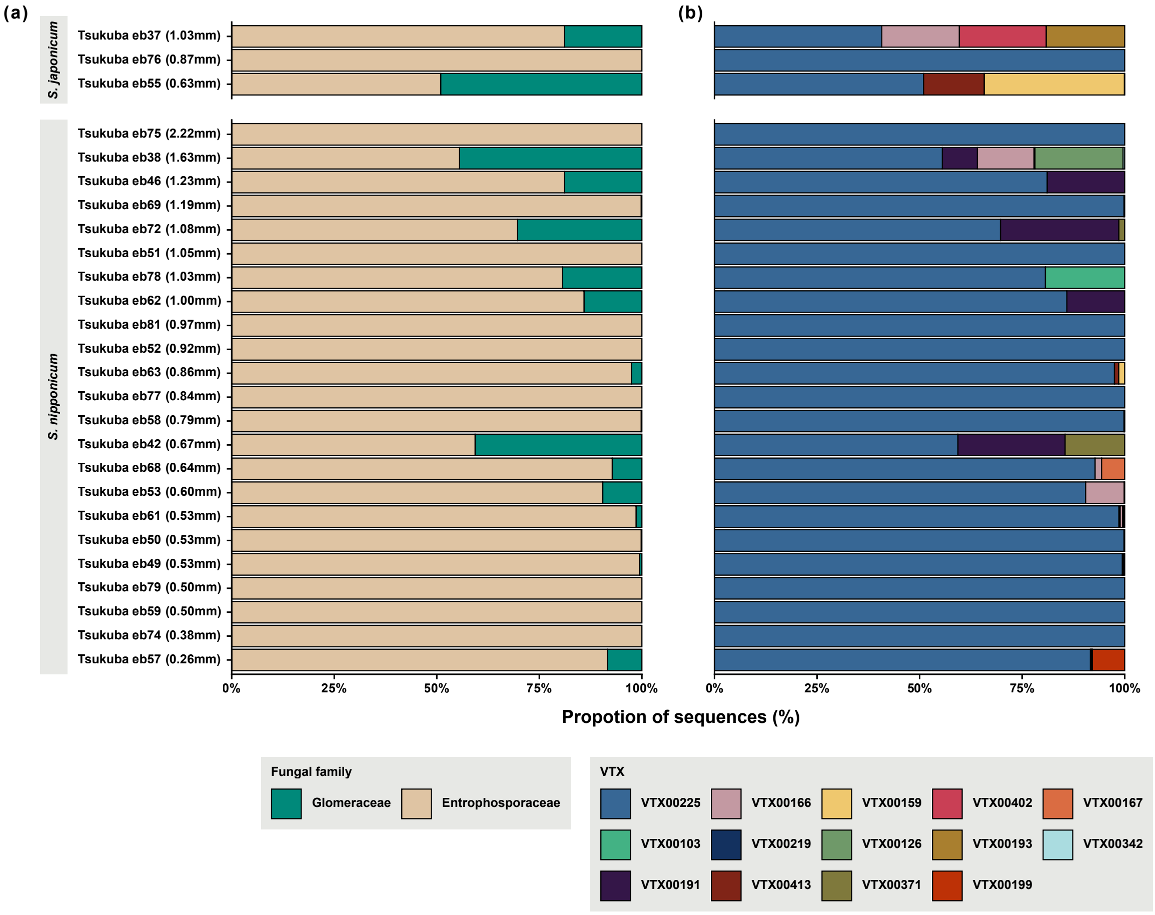

Figure S6. Relative abundance of mycorrhizal communities associated with each *Sceptridium* gametophyte, including information on its length.

Method S1 Molecular identification of *Sceptridium* gametophytes

Given the sympatric presence of *S. japonicum* and *S. nipponicum*, and the difficulty in distinguishing the two species based on gametophyte morphology, we identified *Sceptridium* gametophytes collected from the Tsukuba population using MIG-seq analysis, a type of reduced representation sequencing technique (Suyama & Matsuki, 2015). After cleaning the samples to remove external contaminants, DNA was extracted using the Qiagen DNeasy® Plant Mini Kit. A MIG-seq library was prepared for 26 *Sceptridium* gametophytes from the Tsukuba population, 2 and 9 *S. japonicum* sporophytes from the Tsukuba and Kobe populations, respectively, and 2 *S. nipponicum* sporophytes from the Tsukuba population, following the protocol by Suyama *et al.* (2022). The library was sequenced using an Illumina MiSeq Sequencer with a MiSeq Reagent Kit v3 (150 cycles, Illumina). The raw MIG-seq data have been deposited in the DDBJ Sequence Read Archive (DRA accession number PRJDB19087).

After removing primer sequences and low-quality reads, we obtained 6,104,388 high-quality reads (averaging 156,523 ± 4,429 reads per sample) from the original 6,950,122 raw reads (averaging 178,208 ± 5,385 per sample). For *de novo* single nucleotide polymorphism (SNP) discovery, we utilized the Stacks 2.65 pipeline (Rochette *et al.*, 2019) with the following parameters: minimum depth of coverage to create a stack (*m*) set at 3; maximum distance between stacks (*M*) at 2; and number of mismatches allowed between sample loci while building the catalog (*n*) at 2. Using the 'populations' module in Stacks, we filtered out SNP sites with high heterozygosity (Ho ≥ 0.6) and those with fewer than three minor alleles. Furthermore, PLINK 1.90 (Chang *et al.*, 2015) was used to remove SNPs in linkage disequilibrium with the parameter set as "--indep-pairwise 50 10 0.1".

To determine gametophyte identity, we employed three analytical techniques: SNP-based maximum likelihood phylogeny, Neighbor-Net network analysis, and STRUCTURE analysis. SNPs not present in at least 50% of the samples were excluded (parameter $R = 0.5$ in 'populations'), resulting in 586 retained SNPs across 39 samples. Maximum likelihood phylogeny was reconstructed using RAxML 8.2.10 (Stamatakis, 2014), applying the GTR substitution model with Lewis' ascertainment bias correction and conducting 1,000 bootstrap iterations. A Neighbor-Net network was constructed using SplitsTree4 4.14 (Huson & Bryant, 2006), based on the uncorrected *P* distance matrix, with ambiguous sites disregarded.

Population structure was evaluated using the ADMIXTURE software 1.3.0 (Alexander & Lange, 2011) with default parameters, across *K* values ranging from 1 to 10, and with cross-validation enabled. The results were visualized using CLUMPAK (Kopelman *et al.*, 2015).

References

Alexander DH, Lange K. **2011**. Enhancements to the ADMIXTURE algorithm for individual ancestry estimation. *BMC Bioinformatics* **12**: 246.

Chang CC, Chow CC, Tellier LC, Vattikuti S, Purcell SM, Lee JJ. **2015**. Second-generation PLINK: rising to the challenge of larger and richer datasets. *Gigascience* **4**: 7.

Huson DH, Bryant D. **2006**. Application of phylogenetic networks in evolutionary studies. *Molecular Biology and Evolution* **23**: 254–267.

Kopelman NM, Mayzel J, Jakobsson M, Rosenberg NA, Mayrose I. **2015**. CLUMPAK: a program for identifying clustering modes and packaging population structure inferences across K. *Molecular Ecology Resources* **15**: 1179–1191.

Rochette NC, Rivera-Colón AG, Catchen JM. **2019**. Stacks 2: Analytical methods for paired-end sequencing improve RADseq-based population genomics. *Molecular Ecology* **28**: 4737–4754.

Stamatakis A. **2014**. RAxML version 8: a tool for phylogenetic analysis and post-analysis of large phylogenies. *Bioinformatics* **30**: 1312–1313.

Suyama Y, Hirota SK, Matsuo A, Tsunamoto Y, Mitsuyuki C, Shimura A, Okano K. **2022**. Complementary combination of multiplex high-throughput DNA sequencing for molecular phylogeny. *Ecological Research* **37**: 171–181.

Suyama Y, Matsuki Y. **2015**. MIG-seq: an effective PCR-based method for genome-wide single-nucleotide polymorphism genotyping using the next-generation sequencing platform. *Scientific Reports* **5**: 16963.
